# Frequency of Esophageal Eosinophilia in a Pediatric Population from Central Brazil

**DOI:** 10.1038/s41598-018-23178-9

**Published:** 2018-03-22

**Authors:** Daniel Strozzi, Marco Aurélio Silveira Botacin, Marilia Adriano Mekdessi, Luciana Ximenes Salustiano, Pedro H. de Paula Silva, Lysa Bernardes Minasi, Gesmar Rodrigues Silva Segundo, Aparecido Divino da Cruz

**Affiliations:** 1 Departamento de Medicina, Programa de Pós-Graduação em Ciências Ambientais e Saúde, Pontifícia Universidade Católica de Goiás, Goiânia, Brazil; 2 Programa de Pós-Graduação Mestrado em Genética, Pontifícia Universidade Católica de Goiás, Goiânia, Brazil; 3 Núcleo de Pesquisas Replicon, Departamento de Biologia, Pontifícia Universidade Católica de Goiás, Goiânia, Brazil; 4 Departamento de Ciências Biológicas, Instituto Federal de Educação, Ciência e Tecnologia do Estado de Goiás (IF Goiano), Rio Verde, Brazil

## Abstract

Here we report a retrospective cross-sectional study on Esophageal eosinophilia (EsEo) frequency in Brazil, for 2, 425 pediatric patients with symptoms associated with gastroesophageal diseases in 2012. EsEo is defined by ≥15 eosinophils per high power field (400x) and confirmed through histological analyses of esophageal biopsies. Overall, 126 patients had EsEo equating to a frequency of 5.2%. There was a significant difference between the endoscopic features of patients with EsEo, where 10.7% had erosive esophagitis, 3.0% had non-erosive esophagitis and 1% showed normal esophageal mucosa. According to the interaction of the variables in the Classification and Regression Tree Analysis, most patients diagnosed with EsEo were older males with erosive esophagitis. On the other hand, the lowest frequency of EsEo was found among younger females with non-erosive esophagitis/normal mucosa. Environmental conditions, including climate variation and changes, were observed in association with EsEo, supporting a potential role for environmental factors in its pathogenesis. There was an inverse correlation between the number of EsEo, rainfall and humidity. EsEo is a relatively frequent finding in the pediatric population of Brazil with symptoms of gastroesophageal diseases. Both clinical and histological examinations of patients are important for a reliable diagnostic of EsEo cases and to patient care.

## Introduction

Esophageal eosinophilia (EsEo) is a chronic inflammation characterized by the increase of eosinophil infiltration limited to the eophageal epithelium^1^. EsEo has become more frequently diagnosed in patients undergoing upper digestive endoscopy (UDE) for symptoms associated with gastroesophageal diseases^2^. EsEo has a multifactor etiology, including food allergies^3^, atopy^4^, and genetic^5^ and environmental factors^6^. The major causes of isolated EsEo are the gastroesophageal diseases, namely eosinophilic esophagitis (EoE), proton pump inhibitor responsive esophageal eosinophilia (PPI-REE) and gastroesophageal reflux disease (GERD), for all of which the clinical diagnoses are different and require specific treatments^7,8^. According to Dellon *et al*. the aforementioned gastroesophageal diseases cannot be distinguished by the eosinophil count or other associated morphological features^8^.

EsEo occurs in both adults and children. However, the symptoms vary with age^9^. The diagnosis of the eosinophilic inflammation of the esophagus is dependent on the evaluation of both clinical symptoms and histological analysis of esophageal biopsies^8^. EoE and PPI-REE are the main esophageal diseases in patients diagnosed with EsEo. For both diseases, esophageal biopsies present ≥15 eosinophils per high-powered field (HPF) in histological analysis^10^.

Taking into consideration that EoE as the main condition having EsEo, epidemiological data have suggested variable incidence rates of the disease in different parts of the world. In Australia, it was reported an 18-fold increase in EoE cases^11^, while in the USA, it was reported a 70-fold increase in the number of cases of EoE in the last 15 years^12^. Nevertheless, the frequency of EoE has not been fully determined worldwide. The estimated frequency of EoE varies between 2 and 10% for the pediatric patients undergoing gastroesophageal endoscopy with symptoms related to gastroesophageal diseases^13^. In Brazil, a few studies have reported on pediatric EoE, analyzing isolated cases or case series^14–17^.

To date, there has not been any report of a study focused in a large cohort undergoing endoscopy in Brazil. Thus, here we report on the first retrospective cross-sectional study on the frequency of EsEo in central Brazil, within a large population of pediatric patients with symptoms associated to gastroesophageal diseases. First, the histological features of esophagus biopsies were analyzed to determine the number of eosinophils per high power field (HPF). Then, the variables sex, age, and endoscopic results of patients were compared with the frequency of cases diagnosed with EsEo. Finally, the annual fluctuation of rainfall, relative humidity, and temperature were correlated with the number of cases diagnosed to determine the influence of relevant environmental variations on the frequency and pathogenesis of EsEo.

## Results

The endoscopic medical records and biopsies of 2,425 patients were evaluated in the current study. Sex composition of the studied population was as follows 51.3% and 48.7% were female and male patients, respectively. With respect to age, participants distributed in groups where 18.7% were in 0 to 2 years old, 32.1% in 3 to 6 yo, 27.8% in 7 to 10 yo, and 21.5% 11 to 15 yo.

The endoscopic diagnostics for all patients included 36.8% with erosive esophagitis, 37.1% with non-erosive esophagitis, and 26.1% with normal esophageal tissue. From the cohort of 2,425 patients, 126 patients were diagnosed with EsEo (e.g. had ≥15 eosinophils per HPF in the biopsies of the esophagus), which equates to an overall frequency of 5.2%. The average number of eosinophils per HPF in the esophagus biopsies was 33.8 ± 15.2 with a range of 15 to 100 (n = 126). The average number of eosinophils per HPF in the duodenum was 10.3 ± 0.94 with a range of 10–15 (n = 126). Nine patients were excluded from the initial cohort because their histological reports indicated ≥15 eosinophils per HPF in the duodenal biopsies, which may characterize a systemic eosinophilic disease instead^18^.

The Chi-square test determined the significance of each independent variable separately. With respect to the sex of patients, 2.9% (36 from 1243) of female patients had EsEo while male patients with EsEo presented 2.5 times the number of cases (7.6%–90 from 1182). Thus, it was observed a significant difference (p < 0.001) between the distribution of EsEo among male and female patients. For the age groups, 3.1% (14 from 453) of patients in the age group 0–2 had EsEo, 6.2% (48 from 778) for the 3–6, 5.6% (38 from 673) for the 7–10 and 5% (26 from 521) for the age group of 11–15. Furthermore, Table 1 demonstrate the distribution of frequency between sex and age of all patients with EsEo. In addition, 10.7% (92 of 893) of patients with erosive esophagitis, 3% (27 from 889) with non-erosive esophagitis and 1.1% (7 of 633) with a normal endoscopic appearance had ≥15 eosinophils per HPF (Table 2).

**Figure 1:**
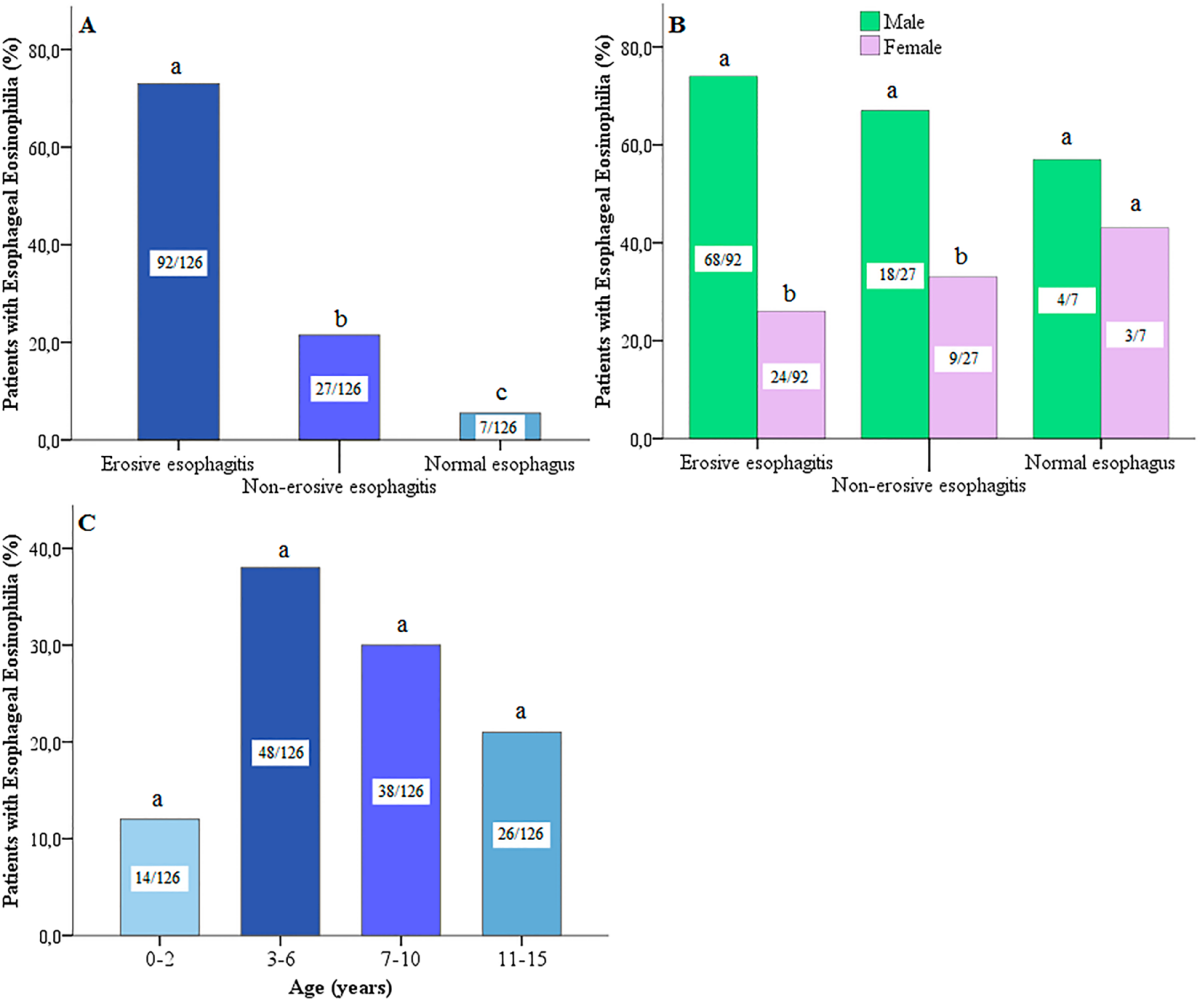
Percintage of patients with esophageal eosinophilia for each independent variable. (**A**) Endoscopic diagnostic, (**B**) Endoscopic diagnostic x Sex and (**C**) Age. Different letter denotes a significance level of 5% (p < 0.001, Pearson’s Chi-squared post-hoc).

There was also a significant difference (p < 0.001) between patients with EsEo in regards to the endoscopic diagnostic. EsEo was about 3x more common in patients with erosive esophagitis (92/126; 73%) when compared to non-erosive esophagitis (27/126; 21.4%) and patients with a normal endoscopic appearance (7/126; 5.6%) as shown in Fig. 1A. In addition, within the patients with erosive esophagitis, 74% were male and 26% were female. The same trend was observed for the patients with non-erosive esophagitis with 67% male and 33% female patients (Fig. 1B). However, no differences in sex were observed for patients with a normal endoscopic appearance. Similarly, there were no significant differences between patients with EsEo amongst the different age groups (p = 0.117, Fig. 1C), when the variable sex was analyzed separately.

**Table 1:** Frequency table with sex and age of all patients that underwent upper digestive endoscopy for symptoms of gastroesophageal diseases in a tertiary care service in Goiânia, Brazil. EsEo = Esophageal Eosinophilia.

Sex		Male			Female			Combined sexes		
Histological analysis		EsEo			EsEo			EsEo		
Age (years)		No	Yes	Total	No	Yes	Total	No	Yes	Total
0–2	Count	217	9	226	222	5	227	439	14	453
	Frequency (%)	96.0	4.0	100	97.8	2.2	100	96.9	3.1	100
3–6	Count	368	35	403	362	13	375	730	48	778
	Frequency (%)	91.3	8.7	100	96.5	3.5	100	93.8	6.2	100
7–10	Count	287	27	314	348	11	359	635	38	673
	Frequency (%)	91.4	8.6	100	96.9	3.1	100	94.3	5.7	100
11–15	Count	220	19	239	275	7	282	495	26	521
	Frequency (%)	92.0	8.0	100	97.5	2.5	100	95.0	5.0	100

**Table 2:** Frequency table with the endoscopic diagnostic of all patients that underwent upper digestive endoscopy for symptoms of gastroesophageal diseases in a tertiary care service in Goiânia, Brazil. EsEo = Esophageal Eosinophilia.

Endoscopic diagnostic	Histological analysis			Total
	EsEo	No	Yes	
Erosive esophagitis	Count	801	92	893
	Frequency (%)	89.3	10.7	100
Non-erosive esophagitis	Count	872	27	899
	Frequency (%)	97.0	3.0	100
Unaffected esophagus	Count	626	7	633
	Frequency (%)	98.9	1.1	100

**Figure 2:**
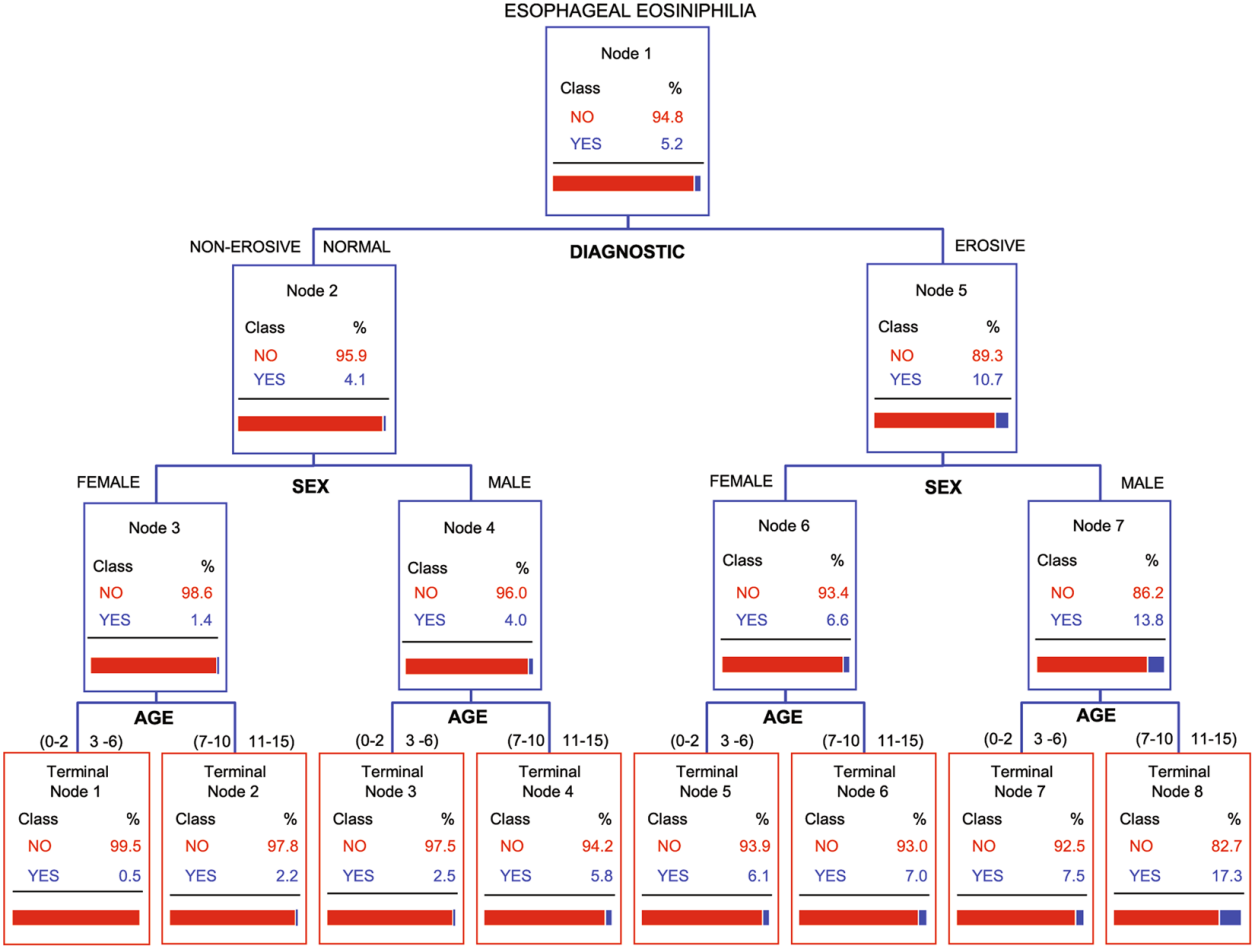
Univariate classification and regression tree explaining 77% of the variability in the model. The CART model is based on the dependent variable esophageal eosinophilia and independent variables endoscopic diagnostic, sex and age. The bars in the bottom of each node represents the frequency of esophageal eosinophilia cases within the interaction.

**Figure 3:**
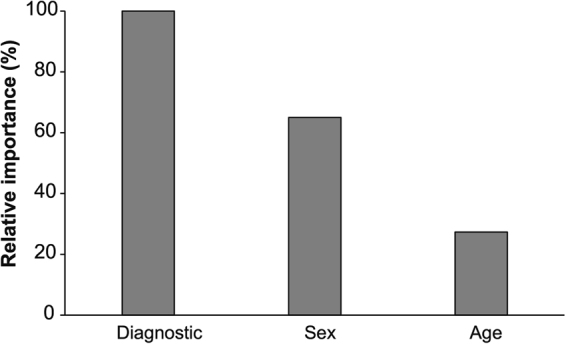
Relative importance of all independent variables derived from the CART model.

Overall, the CART model explained 77% of the variability in the data (Fig. 2). The root node (Node 1) determined the frequency of EsEo amongst all patients (5.2%). The CART analysis identified the endoscopic diagnostic as the most important independent variable (nominally 100%; Fig. 3). This variable was also the first split in the tree, separating the non-erosive esophagitis and the normal endoscopic appearance (Node 2) from the erosive esophagitis (Node 5) in two homogenous groups (Fig. 2). The sex of patients was subsequently the most important variables (65%, Fig. 3), where male patients (Nodes 4 and 7) had always a higher frequency of EE than the female patients (Nodes 3 and 6, Fig. 2), within the respective endoscopic diagnostic groups. The age group of patients had the lowest relative importance in the CART model (27.3%, Fig. 3). For this variable, regardless of the interaction of endoscopic diagnostic and sex, the older age groups (7–10 and 11–15) had a higher frequency of EsEo than the younger age groups (0–2 and 3–6, Fig. 2).

According to the CART model, the highest overall frequency of EsEo occurred in male patients, aged between 7–15 years with an erosive esophagitis endoscopic diagnostic (Terminal node 8, Fig. 2). The same trend was observed for the female patients with erosive esophagitis, where the older age group (7–15, Terminal node 6) had a higher EsEo frequency than the younger age group (0–6, Terminal node 5). Conversely, the lowest frequency of EsEo occurred in female patients within the younger age groups (0–6 years) with a non-erosive esophagitis and normal endoscopic appearance of the esophagus (Terminal node 1, Fig. 2). Within the non-erosive esophagitis and normal endoscopic appearance group (Node 2), male patients also had higher frequency of EsEo than the female patients, with again the older age groups (Terminal node 2 and 4) presenting a higher EsEo frequency than the younger age groups (Terminal node 1 and 3, Fig. 2).

**Figure 4:**
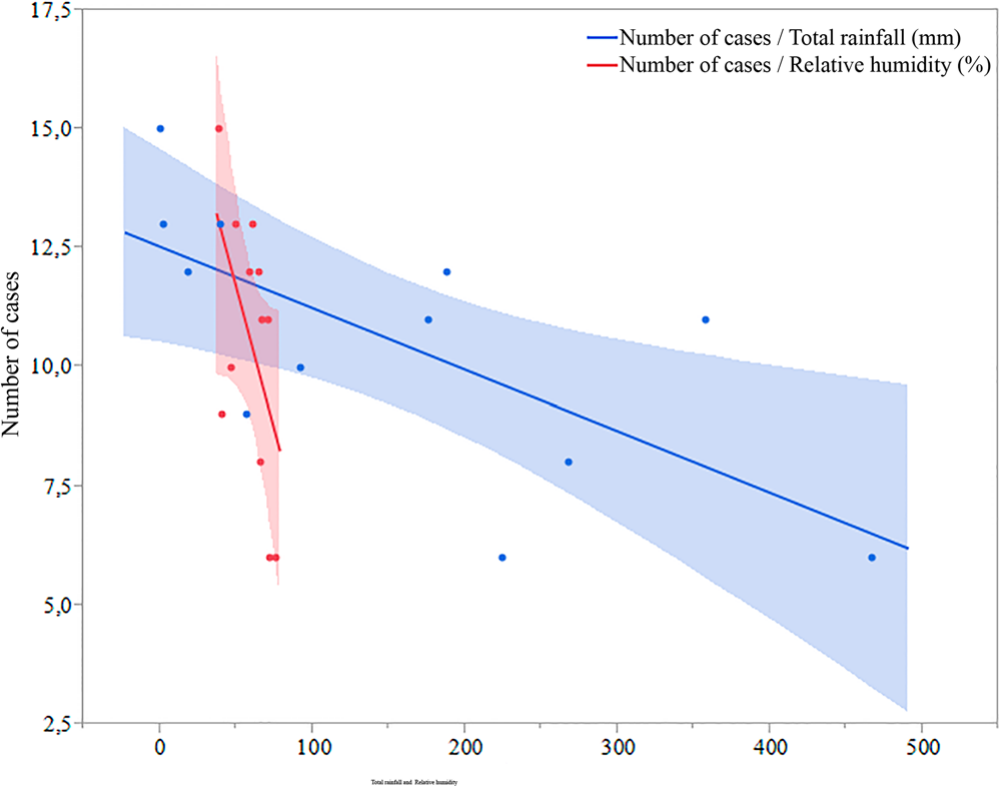
Correlation between total precipitation and number of cases with EsEo from central Brazil.

There were a moderate inverse correlation between the number of EsEo cases and rainfall (r = −0.69; p = 0.01) and between the number of EsEo and humidity (r = 0.58; p = 0.04) determined through Pearson’s correlation analysis. The number of EsEo cases increased when the total rainfall and relative humidity decreased, this period in Central Brazil is represented by the months of May, June, July, and August compared to the higher rainfall and relatively higher humidity present in the others months (Fig. 4). A total of 42,1% (53/126) of all cases of EsEo were diagnosed from May to August 2012 at the beginning of symptoms. No correlation was found between the number of EsEo and the temperature variation for the studied cohort in Central Brazil.

## Discussion

EsEo is a chronic eosinophil infiltration in the esophageal epithelium frequently diagnosed within patients with symptoms of gastroesophageal diseases. Despite the importance and relative high occurrence among pediatric patients, this is the first large study to determine the frequency of EsEo in a pediatric population from central Brazil. The results have shown that the frequency of EsEo is 5.2% for patients with age between 0 to 15 years old. Furthermore, there were significant differences between sex, age, and endoscopic diagnostic of patients. The frequency of EsEo was 2.5 times higher for male than for female patients. Additionally, there was also a significantly higher frequency of EsEo in patients with erosive esophagitis compared with patients with non-erosive esophagitis and normal esophagus endoscopic diagnostic. However, we found that the age of patients with EsEo was only significant when interaction was considered with the sex and endoscopic diagnostic variables, where the frequency of EsEo in older patients (7–15) was higher than in younger (0–6) patients.

The frequency, incidence, and prevalence of EsEo are not well studied, the majority data around EsEo is derived from EoE studies which show highly variable data dependent on both the study design and the population studied. In the current study, we found an EsEo frequency of 5.2% among the children examined, and this frequency is within the range observed worldwide when deriving the data from scientific reports on EoE, considering that EsEo is a histological marker for EoE diagnosis^16^. Moreover, the frequency of EsEo with ages between 0–18 years varies for patients undergoing upper digestive endoscopy. The high variability on types of diet, environmental, and genetic factors encountered in different regions might explain the variation in EsEo frequency worldwide. We found approximately 2.5:1 male to female ratio within our group of children with EsEo. The Chi-squared analysis did not show significance for the age of our patients with respect EsEo distribution. However, significant difference for the age groups was detected after applying the CART analysis. This highlights the importance of using robust and flexible statistical analysis to uncover complex interactions between multiple independent variables contributing to the frequency of EsEo. In the present study, there was a higher presence of EsEo in children between 7 to 15 years compared with younger children (0 to 6). Older children are exposed to a higher diversity of food with allergenic potential, and therefore have a higher risk of developing eosinophilic infiltration in the esophageal mucosa^19^. On the other hand, these confronting results show age alone is not a reliable variable to determine the risk of eosinophil infiltration in the esophageal epithelium of a pediatric population. Thus, Clinicians must be careful with the management of patients of all ages with symptoms of gastroesophageal diseases who must be submitted to a full clinical and histological examination prior to the diagnosis of the gastroesophageal diseases.

The vast majority of patients with EsEo in this study had a positive endoscopic diagnostic for esophagitis. Additionally, over 70% of patients had erosive esophagitis. However, about 1% of patients with EsEo had a normal esophagus upon endoscopic examination in the current study. The careful follow-up of patients with EsEo requires especial attention to the clinical symptoms despite a normal appearance of the esophageal mucosa as esophagitis, dysphagia or food impaction might exist.

Environmental conditions, including climate variation and changes, are important factors in the pathogenesis of EsEo^20^. In the central region of Brazil, we found a negative correlation between the number of EsEo cases and the monthly rainfall over the year when the cases were diagnosed. Temperature variation was not important in EsEo development in the region, mainly because there were very little changes in the average temperature throughout the year, which varied from 23.3 to 28.0 °C (data not shown). Hurrell *et al*. have reported that cold and arid regions have a higher prevalence of EsEo than regions with a tropical climate^21^, which is in agreement with our findings as 1:2 cases of EsEo was diagnosed during the drought season in central Brazil, ranging from May through September. The cooler and drier weather conditions promoted respiratory and atopic diseases associated with EsEo^20^. Thus, there was almost a 40% increment (t = 7,95; p, 0,001) in the diagnosis of EsEo in the winter season, which is an important and useful observation for clinical practitioners. In Brazil, it is noteworthy to mention school vacations are scheduled for January and July. Thus, if the diagnoses of EsEo were based on health checkups at the holiday seasons, than the numbers of cases in January should be similar to July, which was not observed in the current study.

In conclusion, we have demonstrated that EsEo is frequently found in a pediatric population in central Brazil with symptoms of gastroesophageal diseases, suggesting higher prevalence for this particular group of diseases in the country. It is important to focus on prospective studies about potential molecular and genetic mechanisms involved in the pathogenesis of EsEo to distinguish the different gastroesophageal diseases (EoE, PPI-REE and GERD), as well as understanding the sex differences subjacent to the development of EsEo. In this context, the diagnosis and treatment of patients would be improved and at last provide a better quality of life to patients with EsEo.

## Methods

### Study and patients

This was a retrospective, cross-sectional study on the frequency of EsEo in a pediatric population from central Brazil with symptoms of gastroesophageal diseases. Overall, the medical records of 2.434 patients were evaluated, however nine patients were excluded (see below in the Results section). Thus, 2,425 patients between ages 0 and 15 years were included in the studied population. All participants underwent UDE from January to December 2012 in a tertiary service in Goiânia-Goiás, Brazil. The most common indications for performing an UDE were recurrent abdominal pain and vomiting, food refusal and dysphagia.

The study protocol was approved and authorized by the ethical committee of research on humans from the Pontifícia Universidade Católica de Goiás (PUC Goiás), under the identification code #36241914.1.0000.0037, following guidelines and criteria of the resolution number 466/2012-CONEP from the Brazilian National Health Council and the principles of the Declaration of Helsinki. Data from medical reports were reviewed anonymously and all privacy requirements were met. Informed consent was obtained from all participants and/or their legal guardians by their assistant physicians to be included in this study and to share data and information from the endoscopy and histological analysis.

### Upper digestive endoscopy and histology of biopsies

All patients underwent UDE procedures followed by tissue biopsies of the esophagus. The endoscopic appearance of the esophageal mucosa was graded as erosive esophagitis, non-erosive esophagitis, and normal tissue. The endoscopic appearance of the oesophageal mucosa was graded as normal endoscopic appearance grade 0, non-erosive esophagitis grade 1 according to Hatzel *et al*.^22^ and erosive esophagitis according to the Los Angeles Classification^23^.

The biopsies were prepared for histological analysis using a Histokinette T/P800^©^ (American Optical Co., USA) and embedded in paraffin using a Tissue-Tek^©^ II system (Sakura Finetek Inc., USA). The sections were sliced using an autocut microtome 2040^©^ (Reichert-Jung, USA) and stained in hematoxylin-eosin solution. Microscopic analyses of the tissue samples were made using a light microscope (Olympus, USA) at 400x magnification. In the current study, a positive diagnosis of EsEo was reached if the inflammation in biopsy specimens showed ≥15 eosinophils per HPF, according to the guidelines adopted by the American Gastroenterology Association (AGA) and the North American Society of Gastroenterology (NASG). Three biopsies were taken from each patient from the upper, mid and lower oesophagus. All 126 cases of EsEo who had positive biopsies, i.e. >15 eosinophils per HPF, were further analyzed by two other independent pathologists to confirm the diagnosis of EsEo.

### Statistical analysis

The frequency and descriptive statistics were first calculated. Subsequently, we analyzed each independent variable (sex, age, and endoscopic diagnostic) separately against the dependent variable (EsEo) using the Pearson’s Chi-squared test and *post-hoc* comparisons were performed for the independent variables with significant differences. The p values < 0.05 were considered significant. The descriptive statistics and Chi-squared analyses were performed using the IBM SPSS Statistics software version 20 (Chicago, USA).

A classification and regression tree (CART) was used to analyze the interaction of all independent variables on the frequency of EsEo (Salford Predictive Modeler software - SMP V7.0, USA). This analysis also identified the relative importance of each independent variable in the CART model. Finally, to determine the relationship between the data of EsEo diagnosis and the variation of monthly precipitation, mean temperature, and relative humidity in 2012, a Pearson’s correlation was performed. Climate variation for the studied period in Brazil was publically available at http://www.inmet.gov.br/portal. The amount of rainfall, temperature, and relative humidity were assessed for the year 2012 at the BDMEP (Banco de Dados Meteorológicos para Ensino e Pesquisa) from the INMET (Instituto Nacional de Meteorologia) of the Ministério da Agricultura, Pecuária e Abastecimento.
